# Non-Intensive Care Unit Acquired Pneumonia: A New Clinical Entity?

**DOI:** 10.3390/ijms17030287

**Published:** 2016-02-25

**Authors:** Marta Di Pasquale, Stefano Aliberti, Marco Mantero, Sonia Bianchini, Francesco Blasi

**Affiliations:** 1 Department of Pathophysiology and Transplantation, Università degli Studi di Milano, IRCCS Fondazione Ospedale Maggiore Policlinico Cà Granda, Milan 20122, Italy; marta.dipasquale@gmail.com (M.D.P.); mantero.marco@gmail.com (M.M.); 2 School of Medicine and Surgery, University of Milan Bicocca, AO San Gerardo, Via Pergolesi 33, Monza 20090, Italy; stefano.aliberti@unimib.it; 3 Pediatric Highly Intensive Care Unit, Department of Pathophysiology and Transplantation, Università degli Studi di Milano, Fondazione IRCCS Cà Granda Ospedale Maggiore Policlinico, Milan 20122, Italy; sonia.bianchini@unimi.it

**Keywords:** nosocomial infection, hospital acquired pneumonia, risk factors, prevention, microbiology

## Abstract

Hospital-acquired pneumonia (HAP) is a frequent cause of nosocomial infections, responsible for great morbidity and mortality worldwide. The majority of studies on HAP have been conducted in patients hospitalized in the intensive care unit (ICU), as mechanical ventilation represents a major risk factor for nosocomial pneumonia and specifically for ventilator-associated pneumonia. However, epidemiological data seem to be different between patients acquiring HAP in the ICU *vs.* general wards, suggesting the importance of identifying non ICU-acquired pneumonia (NIAP) as a clinical distinct entity in terms of both etiology and management. Early detection of NIAP, along with an individualized management, is needed to reduce antibiotic use and side effects, bacterial resistance and mortality. The present article reviews the pathophysiology, diagnosis, treatment and prevention of NIAP.

## 1. Search Strategy and Selection Criteria

We searched Medline for papers published from 1 January 2000 to 31 October 2015. We used the search terms “hospital-acquired pneumonia” or “nosocomial pneumonia”, in combination with the terms “epidemiology”, “diagnosis”, “aetiology”, “pathophysiology”, “risk factors”, “management”, “treatment”, “outcomes”, “prevention”, and their variations. We restricted the search strategy to adults. We also searched the reference lists of articles identified by this search strategy. Review articles and book chapters are cited to provide readers with more details and more references. Among studies found with this search strategy, we included only those including patients with hospital-acquired pneumonia according to American Thoracic Society/Infectious Diseases Society of America ATS/IDSA guidelines criteria, and who were hospitalized in both medical and surgical wards. Studies on patients hospitalized in intensive care units were excluded. Studies providing data from a mixed population (both intensive care units and general wards patients) were also excluded.

## 2. Introduction

Hospital-acquired pneumonia (HAP) is the second most important cause of nosocomial infections and accounts for high morbidity and mortality worldwide [1,2,3]. HAP also carries a relevant economic burden on healthcare services and resources [4,5,6].

HAP is currently defined as pneumonia occurring at least 48 h after hospital admission and did not appear to be incubating at the time of admission [2,6]. “HAP—Nosocomial pneumonia” specifically identifies three different types of pneumonia: (1) ventilator-associated pneumonia (VAP), defined as pneumonia developing more than 48 to 72 h after initiation of mechanical invasive ventilation; (2) non ventilator-associated pneumonia in patients hospitalized in intensive care unit (ICU) (NV-ICUAP); and (3) nosocomial pneumonia occurring in patients hospitalized in general wards outside of the ICU (non ICU-acquired pneumonia -NIAP). Health-care associated pneumonia (HCAP) will not be considered in this review according to recent microbiological data and the latest recommendations published by the European Respiratory Society and the European Society of Clinical microbiology and Infectious diseases [7,8,9].

**Figure 1:**
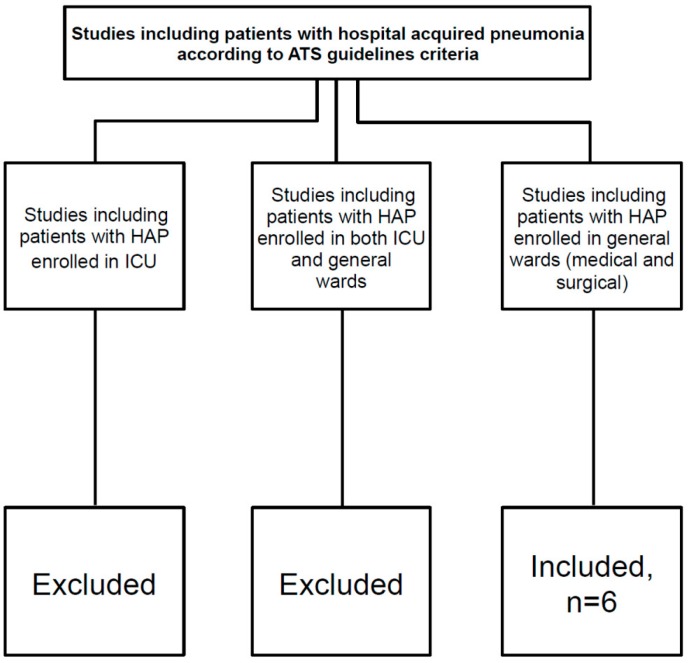
Inclusion and exclusion criteria used during the literature search. ATS American Thoracic Society, HAP hospital acquired pneumonia, ICU intensive care unit.

The vast majority of studies on HAP included patients hospitalized in the ICU (either NV-ICUAP or VAP). Evidence-based data on NIAP are largely extrapolated from experiences coming from the ICU setting. Nevertheless, the absence of intubation and invasive mechanical ventilation could differentiate the etiology and physiopathology of NV-ICUAP/VAP *vs.* NIAP, suggesting that the microbiology and therapeutic approaches in these two diseases might be different. Thus, there is a clear need of reviewing the available data on NIAP, in order to better identify, treat and eventually prevent the development of this specific disease [10,11,12,13,14,15], see Table 1. We included studies specifically enrolling adult patients with HAP hospitalized in general wards. Studies performed in the ICU or including a mixed population of ICU and ward patients were excluded, see Figure 1.

The purpose of this review is to evaluate current evidence on pathogenesis, diagnosis, treatment, and prevention of NIAP and to identify specific research needs in this field.

## 3. Epidemiology

The global incidence of HAP is around 5–10 episodes per 1000 hospital admissions [3] and it is higher among patients requiring mechanical ventilation, ranging between 10 and 15 cases per 1000 ventilator days [2]. The incidence of NIAP has not been fully evaluated in literature in light of a lack of detection of cases in general wards, absence of defined diagnostic protocols and the difficulty in obtaining samples for etiological diagnosis. Most of the available data come for studies enrolling a mixed population of patients who developed a nosocomial pneumonia either in the ICU or general wards (incidence ratio of 0.5–2 per 100 patients) or those who underwent thoracic or abdominal surgery (incidence ratio of 3.8–17.5 per 100 patients), immunosuppressed patients (incidence ratio of 19.5–20 per 100 patients) or the elderly (incidence ratio of 0.7–1.7 per 100 patients) [16]. The incidence of NIAP reported from the few studies conducted only in patients admitted to the general wards ranges from 1.6 to 3.67 cases per 1000 admissions [10,11,12,13,14,15].

Together with other types of HAP, NIAP is the most frequent cause of death among nosocomial infections worldwide. Mortality rates range from 20% to 70%, depending on patient underlying conditions and causative pathogen [10,11,12,13,14,15].

## 4. Risk Factors for NIAP

The most common risk factors for NIAP include advanced age, the presence and severity of underlying diseases, such as chronic renal failure, anemia, neoplasm and chronic obstructive pulmonary disease (COPD), malnutrition, depression of consciousness and nosocomial infections or hospital admissions in the previous month, see Table 2 [10,11,15]. These characteristics increase the patients’ risk of aspiration of contaminated respiratory or gastric secretions and decrease patients’ defenses. A study identified serum albumin level as an independent predictor of HAP in patients with stroke, confirming patient’s nutritional status as an important feature that should be evaluated in hospitalized patients to avoid nosocomial pneumonia [17].

Beyond risk factors related to patient’s performance status, other risk factors for NIAP could be identified, including therapeutic procedures, such as thoracic or upper abdominal surgery, the use of nasogastric tubes, immunosuppressive treatment, previous antibiotic treatment, the length of hospitalization and the use of acid suppressive medication [10,11,18]. Notably, the association with HAP seems to be more significant for proton-pump inhibitors than histamine-2 receptor antagonists [18].

Risk factors for increased mortality include severe underlying conditions, bilateral involvement, the presence of sepsis, respiratory failure or multi-organ failure, an inappropriate initial antimicrobial therapy and infections due to multi-drug resistant (MDR) microorganisms [10].

## 5. Physiopathology

**Figure 2:**
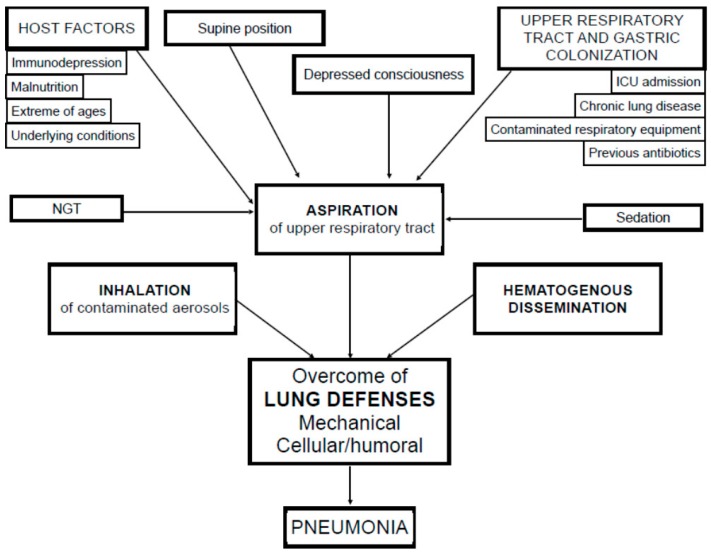
Pathogenesis. ICU intensive care unit; NGT nasogastric tube.

Different mechanisms may contribute to the development of HAP, such as aspiration of upper respiratory tract secretions, inhalation of contaminated aerosols and, more rarely, haematogenous dissemination from septic foci, see Figure 2 [19]. Previous studies clearly recognized microaspiration of contaminated oropharyngeal or gastric secretions (colonized by pathogenic bacteria) as the main causative mechanism [3,4,5,8]. Subclinical microaspiration may be induced by depressed consciousness, disorders in swallowing and cough reflex, or alterations in gastrointestinal motility. Microorganisms in the oropharyngeal flora are the cause of pneumonia. After a few days from hospital admission, bacterial flora shifts to Gram-negative bacilli, especially if patients are malnourished, severely ill or already under antibiotic treatment [2,10]. Furthermore, the inhalation of aqueous (showers and water taps) or airborne aerosols (dust or saliva drops) has been identified as possible, although uncommon, cause of pneumonia sustained by specific microorganisms such as *Legionella* spp., *Aspergillus* spp., *Chlamydia pneumoniae* and virus [3]. Inadequate hand washing by medical personnel could also facilitate the spread of resistant bacteria among patients [19]. Most of these data come from studies that enrolled patients with HAP in general, while specific aspects of NIAP physiopathology have not been explored yet.

## 6. Causative Pathogens

The pathogens causing NIAP may vary, usually depending on different factors, including time of pneumonia onset, previous patient health status, previous antibiotic therapy, residence in a nursing home and finally the available diagnostic techniques used to identify these microorganisms. As far as we know, etiology of nosocomial pneumonia could be different in NV-ICUAP/VAP *vs.* NIAP. Unfortunately, most of the microbiological data available for nosocomial pneumonia are primarily derived from studies performed in the ICU [3,4], see Table 1.

The oropharyngeal flora of non-ventilated patients admitted to the general wards may remain unaltered for a long period of time, in comparison to what happens in mechanically ventilated patients or those admitted to the ICU. Therefore, NIAP pathogens might mainly resemble those causing community-acquired pneumonia, such as *Streptococcus pneumoniae* and *Staphylococcus aureus* [10,14].

Time of onset is also a crucial factor that deserves to be carefully considered. Early-onset pneumonia is usually caused by community-acquired pathogens such as *Haemophilus influenzae*, *S. pneumoniae*, or methicillin-susceptible *S. aureus* (MSSA) [11]. Pneumonia that develops after five days from hospitalization (“late onset”) is often caused by aerobic Gram-negative bacilli (*P. aeruginosa*, *Enterobacteriaceae*, or *Acinetobacter*) or methicillin-resistant *S. aureus* (MRSA) [2,11].

A careful evaluation of patient risk factors and comorbidities can also help to identify likely causative pathogens of NIAP, see Table 2. Gram-negative bacilli are more prevalent in previously ill patients, reflecting a switch in oropharyngeal flora, and among them the most frequent pathogens are *P. aeruginosa*, *A. baumannii*, *Enterobacteriaceae* (*Klebsiella* spp., *Enterobacter* spp., *Serratia* spp., *etc.*) and *H. influenzae* [10,11,14]. Prior antibiotics and hospitalizations could be more relevant than time of onset [10]. Patients with none of the above characteristics have a much lower risk of harbouring a highly resistant organism [10,11,14]. *P. aeruginosa* is more frequent in patients with severe underlying illness (especially structural lung disease), prolonged hospitalization and wide spectrum antibiotic therapy [7,8]. Moreover, the extensive use of third generation cephalosporins and fluoroquinolones has increased the prevalence of *Enterobacteria* producing extended spectrum betalactamases (ESBLs), such as *Klebsiella pneumoniae*, in the general wards [17,20]. *S. aureus* has been identified as predominant causative pathogen in some studies among patients with NIAP [14,15]. MRSA is usually detected in patients with specific risk factors, such as intravascular catheters, nasal carriage and staying in hospitals and/or departments with a high prevalence of colonisation or infection by these microorganisms [3,18]. *S. pneumoniae* is recognized as a predominant causative agent of nursing home pneumonia [21]. In addition, among the elderly, inadequate oral care and difficult swallowing are known risk factors for aspiration pneumonia and anaerobes [22]. Microorganisms such as *L. pneumophila*, anaerobes and viruses are not routinely tested in NIAP and thus, their prevalence is largely unknown [23]. Legionellosis could be diagnosed in immunocompromised patients or in local hospital outbreaks because of contaminated water supplies. Immunocompromised patients or COPD patients receiving systemic steroids could developed pneumonia by *Aspergillus* spp., caused by inhalation of fungal spores from the environment (dust, furniture and ornamental plants), food products or even water [11,18,24]. Rarely, viruses, *Mycobacterium tuberculosis*, *Mycoplasma pneumoniae* or *Chlamydia pneumoniae* could be causative pathogens of nosocomial pneumonia [14,15,25].

## 7. Clinical Presentation and Diagnosis

The clinical presentation of NIAP is not specific. Patients usually present with fever and respiratory symptoms, such as new onset or worsening cough and expectoration with purulent sputum. Dyspnoea and pleuritic chest pain are less frequent and may be caused by other underlying conditions. Moreover, patients with neurological impairment or severe illness may be unable to manifest or express these symptoms. On physical examination, tachypnoea, rales or bronchial breathing sounds may be present, although their detection may often be difficult in patients with underlying diseases [3,10]. Chest radiography (CXR) demonstrates new or progressive infiltrates, consolidation, cavitation or pleural effusion. Differential diagnosis should be made with other processes which may cause similar clinical and radiological changes, including heart failure, pulmonary embolism, ARDS, atelectasis, alveolar haemorrhage, or lung contusions [3]. Diagnosis of HAP is based on the appearance of a pulmonary infiltrate on CXR at least 48 h after hospital admission, which is not attributable to any other cause. The presence of at least two of the three following clinical features is also required: fever, leukocytosis or leukopenia and/or purulent respiratory secretions [4,18]. However, these criteria are not very sensitive and specific, particularly in the elderly, immunosuppressed patients or those with cardiopulmonary diseases. Moreover, CXR may lead to misdiagnosis, as it lacks sensitivity and specificity, compared to chest CT scan [26].

## 8. Microbiological Evaluation

Etiological diagnosis of NIAP is usually based on results of blood cultures, urinary antigens and sputum or tracheal secretions, because of the inability to perform invasive procedures in most cases. Use of invasive techniques to achieve an etiologic diagnosis is less common among NIAP patients in comparison to those with VAP/NVICUAP. The diagnosis of VAP can be based on either clinical findings or results of invasive methods, such as protected specimen brush (PSB), or bronchoalveolar lavage (BAL) [15]. An invasive strategy based on quantitative bronchoscopic specimen culture improves early survival among VAP patients [27], but it is unknown whether this strategy is beneficial for NIAP patients.

The use of invasive diagnostic strategies for NIAP may have several disadvantages, including high costs and significant morbidity. Non-invasive management of HAP may nevertheless have potentially harmful consequences: inadequate treatment leading to higher mortality risk, patients may receive unnecessary or potentially toxic antibiotics, resistant microorganisms may be selected, and diagnosis of a non-infectious cause of fever and pulmonary infiltration may be delayed [15].

Bronchoscopy with BAL should always be considered in immunosuppressed patients or those who do not improve after 48–72 h of empiric antibiotic treatment. A patient is considered to have pneumonia when culture reveals more than 1 × 10^4^ organisms/mL with standard BAL. It has been shown that invasive procedures lead to less antibiotic overuse [15].

## 9. Biomarkers

Biomarkers of infection and sepsis, such as procalcitonin (PCT) and C-reactive protein (CRP), could be used for the diagnosis and management of respiratory tract infections [28]. They may be applied as rapid and accurate tools to determine the possible presence of infection and, thus, avoid antibiotic overuse. Furthermore, a soluble triggering receptor expressed on myeloid cells (sTREM-1) that is up-regulated in the setting of infection has been shown to have a role in identifying pneumonia. However, further studies are needed before this marker may be employed in routine clinical practice.

## 10. Treatment

A delay in administrating effective antibiotics in patients with suspected NIAP may impact their prognosis. Thus, patients should be treated immediately with antimicrobials without waiting for microbial results, even when the patient is clinically stable [1,2,3,4,5,29].

In order to choose the most effective empirical therapy, clinicians must take into account several factors, including the severity of pneumonia, risk factors associated with specific pathogens and the time of pneumonia onset [30]. In addition, it is crucial to know the predominant pathogens in a specific clinical setting, as well as the local antibiotic susceptibilities. There are significant geographical differences in the frequencies of antimicrobial resistance between some European areas and even within the same country, from one hospital to another. Therefore, pathogen and susceptibility patterns should be regarded primarily as potential indicators of general trends and lead to increased attention to the local epidemiology. It is now established that each antimicrobial treatment policy shows a specific selective pressure. Consequently, each local setting recognizes a specific ecological and resistance patterns due to previous and current policies. Therefore, it is clear that recommendations for initial empiric antimicrobial treatment should acknowledge a sufficient flexibility to allow modifications according to local peculiarities [2], which are usually the result of preventive and treatment strategies and the type of population treated.

**Figure 3:**
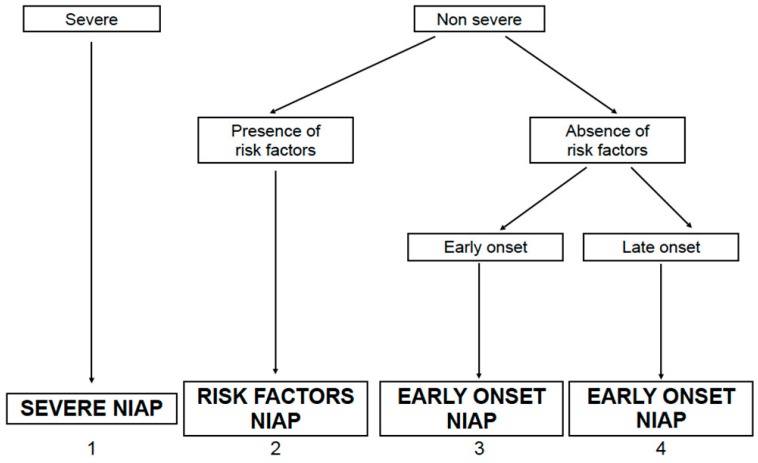
Categories of non ICU acquired pneumonia (NIAP).

According to some authors, four categories of nosocomial pneumonia in non-ventilated patients could be distinguished: (1) severe NIAP; (2) NIAP with risk factors; (3) early-onset NIAP and (4) late-onset NIAP [30], see Figure 3.

**Figure 4:**
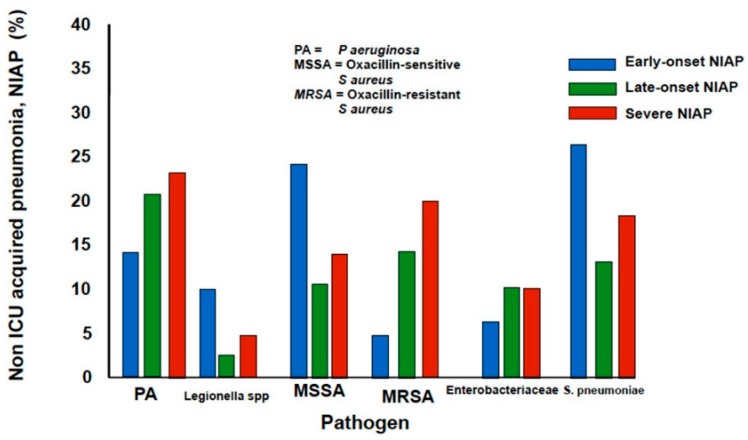
Etiologic pathogens according to categories of non-ICU acquired pneumonia (NIAP).

Severity is defined by the presence of extensive or progressive radiological involvement, respiratory failure, sepsis, and/or severe organ dysfunction. The most frequent pathogens in severe NIAP are *S. pneumoniae*, *P. aeruginosa*, *Enterobacteriaceae*, see Figure 4. Some characteristics could be risk factors for infections by specific pathogens and should be taken into account when deciding empirical antibiotic therapy in patients with moderate-mild NIAP, see Table 2. In patients with none of these risk factors, time of onset of pneumonia is the main variable to guide the empirical therapy.

In case of severe pneumonia or if MDR pathogens are suspected, a combined empirical therapy is suggested in order to prevent the development of resistance during treatment, see Table 3.

### 10.1. De-Escalation and Withdrawal

**Figure 5:**
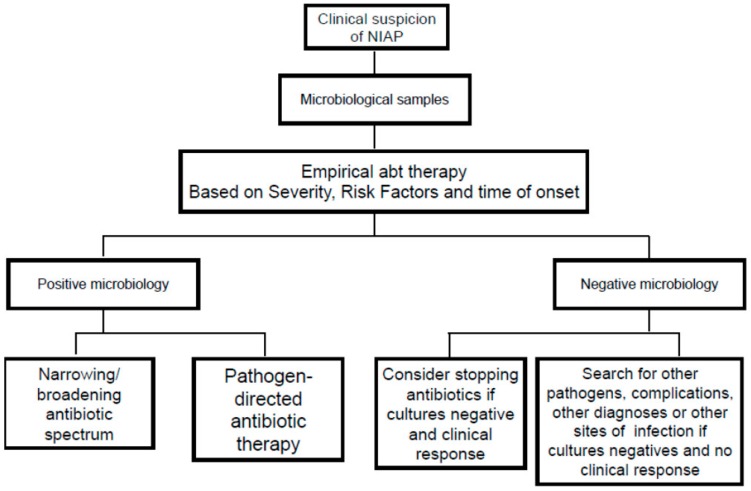
Management strategies for a patient with suspected non ICU acquired pneumonia (NIAP).

Once microbiological results become available, antimicrobial therapy may be modified, either narrowing/broadening the antibiotic spectrum or selecting a pathogen-directed antibiotic therapy or even discontinuing antibiotics if pneumonia is ruled out [3,30], see Figure 5. Experts suggest that treatment could be discontinued if the following three criteria are fulfilled: (1) clinical diagnosis of pneumonia is unlikely (no definite infiltrates on CXR at follow-up and no more than one of the three following findings: temperature >38.3 °C, leukocytosis or leukopenia, and purulent tracheobronchial secretions) or an alternative non-infectious diagnosis is confirmed; (2) tracheobronchial aspirate culture results are non-significant; and (3) there is no evidence of severe sepsis or septic shock [30]. Some studies also indicate that serial measurements of PCT could be a helpful marker in deciding to discontinue antibiotics and, thus, reducing antibiotic use. However, further studies are needed to validate these data [31].

### 10.2. Response to Treatment

Treatment should be considered effective in case of remission of fever, reduction in sputum production and purulence, reduction of leukocytosis, improvement of hypoxaemia, and resolution of possible organ dysfunctions within 48–72 h after initiation of antibiotic therapy. Treatment response could be delayed in patients with severe pneumonia, with more virulent pathogens or in more debilitated patients. Finally, treatment failure could be due to several causes, including: (1) non-infectious aetiology; (2) severe pre-existing host conditions (e.g., underlying diseases, older age, chronic lung diseases and immunosuppression); (3) multi-drug resistant (MDR) microorganisms and (4) infectious complications, such as empyema or pulmonary abscess.

### 10.3. Duration of Therapy

Duration of antibiotic therapy is not well defined in patients with lower respiratory tract infection [32,33]. Usually, if pneumonia is sustained by pathogens such as *S. pneumoniae*, *H. influenzae* or methicillin-sensitive *S. aureus*, 7 to 10 days of treatment might be sufficient. On the other hand, pneumonia caused by other resistant microorganisms treatment should be prolonged to at least 14–21 days. However, most of the indications on duration of antibiotic therapy with patients with pneumonia are based on experts’ opinions [32,33].

## 11. Prevention

Several preventing strategies have been developed to reduce both the incidence of nosocomial infections and specifically of nosocomial pneumonia, with subsequent important clinical and financial consequences, see Table 4. Unfortunately, the majority of studies focused on preventing strategies were carried out in ICU patients, with the majority of them being on invasive mechanical ventilation [6]. Thus, specific preventive strategies targeted for NIAP haven’t been developed yet.

General measures for infection control include alcohol-based hand disinfection, the use of microbiologic surveillance, monitoring and early removal of invasive devices and programs to reduce antimicrobial prescriptions [2,16,34,35,36]. Awareness of the physiopathology of nosocomial pneumonia has led to the development of specific preventive strategies, e.g., to reduce oropharyngeal colonization, cross-contamination from other patients and from the environment and, when possible, correct individual risk factors, such as aspiration [35,37,38,39]. The management of malnutrition is also crucial, as hypoalbuminaemia has been identified as a risk factor for HAP [17]. The use of immunosuppressive drugs, wide-spectrum antibiotics and nasogastric tubes should be avoided, when possible. Finally, each centre should have a nosocomial infection control program in order to improve prevention and management of NIAP, as well as other nosocomial infections.

## 12. Conclusions and Research Needs

Most of the available data on nosocomial pneumonia derive from studies on mechanically ventilated patients. In contrast, there are very few data on NIAP. Our review highlighted some important points that need to be developed in further trials and studies, see Table 4.

First of all, the exact incidence of NIAP is unknown, due to the lack of defined diagnostic criteria and insufficient surveillance of this disease in general wards, in comparison to VAP or NV-ICUAP. Clinicians should monitor suspected NIAP in patients hospitalized in general wards, in order to achieve prompt identification of this disease, to evaluate more precisely its incidence and to better describe its extent and impact on both patients’ prognosis and health care system resources. Subsequently, a diagnostic path should be designed, based on clinical, laboratory, radiological and microbiological data, following a multidisciplinary approach and involving clinicians, intensivists, microbiologists, pharmacists, and infection-control professionals. In particular, microbiological protocols should be developed in order to obtain adequate and standardized samples, to rule out or confirm the diagnosis of NIAP and to identify the most likely causative pathogens. Being aware of the most probable causative pathogens could allow clinicians to remove some known risk factors for this disease and to choose the most effective antibiotic strategy. Moreover, de-escalating and shortening the antibiotic therapy are relevant approaches to decrease the emergence of resistant strains and therefore to minimize treatment failure. Finally, the impact of lower respiratory tract infections on medium and long-term outcomes, including cardiovascular events, is well known in community-acquired pneumonia, while data on nosocomial pneumonia and NIAP are urgently needed [40,41].

## Figures and Tables

**Table 1:** Available literature on non ICU-acquired pneumonia (NIAP).

Author	Year of Publication	Study	Study Setting	Number of pts Enrolled	Incidence/Prevalence of NIAP	Most Frequent Pathogens Isolated in NIAP
Sopena *et al.* [10]	2014	Incident case-control study	Spain, 600-bed tertiary hospital	119 cases with NIAP and 238 controls	2.45 cases/1000 hospital admissions	*S. pneumoniae* 13%, *Enterobacteriaceae* 8%, *P. aeruginosa* 3%, MRSA 3%
Herer *et al.* [15]	2009	Randomized control trial	France, 411-bed facility	68 pts with NIAP	/	*Staphylococcus aureus* 25.4%, MSSA 25%, *P. aeruginosa* 19%
Weber *et al.* [14]	2007	Prospective, observational study	USA, a tertiary care academic hospital	556 pts (588 episodes of pneumonia): VAP 309 pts (327 episodes) NIAP 247 pts (261 episodes)	/	Gram-positive cocci (42.59% [MSSA, 13.33%]; [MRSA, 20.37%]), *P. aeruginosa* 9%
Barreiro-Lopez *et al.* [13]	2005	Prospective case-control study	Spain	67 pts with NIAP	3.35 cases/1000 admissions	/
Sopena *et al.* [11]	2005	Multicenter, prospective, observational study	Spain, 12 teaching hospitals	186 patients with NIAP	3 ± 1.4 cases/1000 hospital admissions	*S. pneumoniae*, 9.7%, *Enterobacteriaceae* 5%, *P. aeruginosa* 4%
Everts *et al.* [12]	2000	Prospective observational study	New Zealand, university-affiliated hospital	126 pts with NIAP	6.1 cases/1000 admissions	*Legionella* spp., 13%, MSSA 3%

pts: patients; NIAP non ICU acquired pneumonia, ICU intensive care unit, VAP ventilator associated pneumonia, MSSA methicillin sensitive *S. aureus*, MRSA methicillin resistant *S. aureus.*

**Table 2:** Risk factors of infection with specific pathogens.

Penicillin-Resistant Pneumococci	Age > 65 years	Betalactam-Therapy (last 3 month)	Alcoholism	Immune Suppressive Illness	Multiple Comorbidities
**Gram-negative bacilli**	Chronic underlying disease	Multiple comorbidities	Residence in a NH	Recent antibiotic therapy	
***P. aeruginosa* and multi resistant Gram-negative bacilli**	Wide spectrum antibiotics	Severe underlying disease	Prior broad spectrum abt therapy	Structural lung disease	Corticosteroid therapy
**Legionella**	Hospital potable water	Previous nosocomial Legionellosis	‒	‒	‒
**Anaerobes**	Gengivitis or periodontal disease	Swallowing disorders	Depressed consciousness	Orotracheal manipulation	‒
**MRSA**	Intravascular devices	Nasal carriage	High prevalence	‒	‒
**Aspergillus**	Corticosteroid therapy	Neutropenia	Transplantation	‒	‒

NH nursing home, MRSA methicillin-resistant *S. aureus*.

**Table 3:** Treatment of non ICU-acquired pneumonia (NIAP).

Beta-lactams * + aminoglycoside Or quinolone	SEVERE NIAP NIAP WITH RISK FACTORS FOR *P. aeruginosa* Gram negative bacilli
Antipseudomonal cephalosporin Or Fluoroquinolones	LATE ONSET NIAP <5 days
Beta-lactam/beta-lactamase inhibitor Or Third generation non-pseudomonal cephalosporin: Or Fluoroquinolones	EARLY ONSET NIAP <5 days
Levofloxacin Or azitromycin	*LEGIONELLA SPP:*
Carbapenems Or b-lactam/b-lactamase inhibitor	ANAEROBES
Vancomycin Or Linezolid	MRSA (Methicillin resistant *S. aureus*)
Amphotericyn B desoxicolate Or amphotericyn liposomal Or Voriconazol	*ASPERGILLUS SPP*.

* include: antipseudomonal cephalosporin, antipseudomonal carbapenem and beta-lactam/beta-lactamase inhibitor.

**Table 4:** Overview of NIAP *vs.* VAP/NV-ICUAP.

	NIAP	VAP/NVICUAP
Diagnosis	New infiltrate on CXR after 48 h of hospital admission +: fever, dyspnea, cough and purulent expectoration, leukocytosis or leukopenia	New or worsening infiltrates on CXR after 48 h after initiation of invasive mechianical ventilation/admission to ICU admission + fever worsening of PaO2/FiO2 purulent tracheal secretions leukocytosis or leukopenia
Stratification	Severe NIAP NIAP with risk factors Early onset NIAP Late onset NIAP	Ventilated patient/spontaneously breathing patient Late *vs.* early onset Presence of risk factors
Microbiological tests	Blood cultures Urinary Antigens Sputum Tracheal aspirate	Blood cultures PBS BAL BAS
Etiology	*S. pneumoniae* *Enterobacteriaceae* *P. aeruginosa* *S. aureus* *Legionella pn.*	Early onset: *S. aureus*, *S. pneumoniae*, *H. influenzae*, non-drug resistant GNEB Late onset: MRSA, drug resistant GNEB, *P. aeruginosa*, *A. baumannii*
Empirical therapy	Severe NIAP: b-lactams with an aminoglycoside or fluoroquinolone Early onset NIAP: b-lactam/b-lactamase inhibitor or third generation non-pseudomonal cephalosporin, or fluoroquinolones Late onset NIAP: Antipseudomonal cephalosporin Fluoroquinolones NIAP with risk factors: specific for each organism	Early onset: aminopenicillin plus b-lactamase-inhibitor Or third generation cephalosporin Or quinolones Late onset: antipseudomonal penicillin Or antipseudomonal cephalosporin Or carbapenems Plus quinolone if MRSA suspected Vancomycin Or Linezolid Antimicrobial treatment of pneumonia with risk factors, any onset: specific for each organism
Prevention	Interventions to modify individual risk factors, such as malnutrition, anemia, and risk of aspiration Hand hygiene Isolation of MDR patients	Avoid intubation Semi-recumbent position to decrease aspiration of oropharyngeal secretions Oral hygiene with chlorhexidine Probiotics Specialized endotracheal tubes (subglottic secretion drainage; silvercoated)
In-hospital mortality	One third of cases	20% to 70%, depending on the characteristics of the patient and the microorganism involved

Table legend: NIAP non ICU-acquired pneumonia, VAP ventilator associated pneumonia, NV-ICUAP non ventilator ICU acquired pneumonia, CXR chest X ray, ICU intensive care unit, PBS protected specimen brush, BAL bronchoalveolar lavage, BAS bronchial aspirate, GNEB gram negative Enteric Bacilli, MRSA methicillin resistant *S. aureus*.
